# Building Interdisciplinary Leadership Skills among Health Practitioners in the Twenty-First Century: An Innovative Training Model

**DOI:** 10.3389/fpubh.2015.00221

**Published:** 2015-10-07

**Authors:** Preeti Negandhi, Himanshu Negandhi, Ritika Tiwari, Kavya Sharma, Sanjay P. Zodpey, Zahiruddin Quazi, Abhay Gaidhane, Jayalakshmi N., Meenakshi Gijare, Rajiv Yeravdekar

**Affiliations:** ^1^Indian Institute of Public Health Delhi, Public Health Foundation of India, Gurgaon, India; ^2^Public Health Foundation of India, Gurgaon, India; ^3^Jawaharlal Nehru Medical College, Datta Meghe Institute of Medical Sciences, Wardha, India; ^4^Symbiosis College of Nursing, Symbiosis International University, Pune, India; ^5^Symbiosis International University, Pune, India

**Keywords:** leadership, interprofessional, interdisciplinary, competencies, health professional education

## Abstract

Transformational learning is the focus of twenty-first century global educational reforms. In India, there is a need to amalgamate the skills and knowledge of medical, nursing, and public health practitioners and to develop robust leadership competencies among them. This initiative proposed to identify interdisciplinary leadership competencies among Indian health practitioners and to develop a training program for interdisciplinary leadership skills through an Innovation Collaborative. Medical, nursing, and public health institutions partnered in this endeavor. An exhaustive literature search was undertaken to identify leadership competencies in these three professions. Published evidence was utilized in searching for the need for interdisciplinary training of health practitioners, including current scenarios in interprofessional health education and the key competencies required. The interdisciplinary leadership competencies identified were self-awareness, vision, self-regulation, motivation, decisiveness, integrity, interpersonal communication skills, strategic planning, team building, innovation, and being an effective change agent. Subsequently, a training program was developed, and three training sessions were piloted with 66 participants. Each cohort comprised a mix of participants from different disciplines. The pilot training guided the development of a training model for building interdisciplinary leadership skills and organizing interdisciplinary leadership workshops. The need for interdisciplinary leadership competencies is recognized. The long-term objective of the training model is integration into the regular medical, nursing, and public health curricula, with the aim of developing interdisciplinary leadership skills among them. Although challenging, formal incorporation of leadership skills into health professional education is possible within the interdisciplinary classroom setting using principles of transformative learning.

## Genesis of Innovation Collaborative on Leadership

Leadership is a complex multidimensional concept that has been defined in many different ways. A visionary leader influences the organizational outlook and has the potential to optimize team performance (1). In the healthcare system, leadership skills are essential to work in a dynamic environment for a minimum acceptable level of healthcare in populations exposed to threats from communicable and non-communicable diseases, to meet the needs of an overstretched public health system and the rising costs of providing healthcare, and to resolve the numerical shortage and poor distribution of healthcare providers globally. Young students who have enrolled in academic programs are the future leaders of healthcare systems. Developing leadership skills among them is of vital importance. The recommendations of the Lancet Commission Report (2), discussing three generations of global education reforms, target a multidisciplinary and systemic approach for health professional education. Transformative learning, the third-generation reform with its focus on the development of leadership skills and interdependence in health education, engages the needs and demands of the twenty-first century health professional education. The purpose of such education reform is to produce progressive change agents in healthcare. The *Future of Nursing* Report (3) also strongly focuses on transformative leadership, stating that strong leadership is critical for realizing the vision of a transformed healthcare system.

Health practitioners have made significant contributions globally to health and development over the past century. Interprofessional education sets the stage for teamwork, and its necessity has grown in importance due to transformational educational reforms and the transforming health systems. Recently, attention has been paid to the concept of interdisciplinary teams working together in healthcare. The elements of collaborative interprofessional practice include commitment to power sharing, distributed leadership, and striving for teamwork (4). The core competencies for interprofessional collaborative practice have been reported by an expert panel in 2011 (5). It stresses the importance of a continuous development of interprofessional competencies by health profession students as part of the learning process, so that they enter the workforce ready to practice effective teamwork and team-based care (5). Although contextualized within the clinical care setting, these competencies are equally important for leadership education in general. It is known that individual reported leadership styles are correlated with leadership outcomes at the organizational level (6). Recent work has also evaluated the impact of a case competition-driven model on development of interprofessional competencies among graduate students and provides a model for assessing interprofessional competency development adapted to non-clinical professions (7).

Recently in India, the lack of and need for healthcare practitioners has been discussed persuasively. The education system for health practitioners in India is compartmentalized with strong professional boundaries among the various health providers (medical, nursing, and public health), with poor coordination between these three academic programs. The current health professional education system in India has minimal focus on the formal development of leadership competencies to address the public health needs of the population. To advance the agenda of interdisciplinary leadership, three institutions in India jointly launched an Innovation Collaborative (IC) with the aim of identifying interdisciplinary leadership competencies in healthcare and develop and pilot an interdisciplinary training model relevant to doctors, nurses, and public health practitioners in India.

## Activities of the Innovation Collaborative

Three institutions partnered in this initiative – Public Health Foundation of India, New Delhi (public health institute); the Datta Meghe Institute of Medical Sciences, Sawangi, Wardha (medical school); and the Symbiosis College of Nursing, Pune (nursing school). The Collaborative was commissioned by the Institute of Medicine, Washington, DC, USA, through a systematic, competitive selection process. The Indian IC was the only one selected from Asia and one of four initiatives globally. The main task of the IC was to develop a training model for medical, nursing, and public health practitioners, incorporating the principles of interdisciplinary leadership in their competencies. A core team was formed including members from all three partner institutes. Additionally, a Technical Advisory Group was formed, composed of experts in the field of health professional education, to oversee and provide guidance to the activities of the Collaborative. Regular meetings were held with the TAG members and their guidance was sought on various activities of the initiative.

The initial activity undertaken by the IC was an exhaustive literature search in order to understand the need for and genesis of interdisciplinary leadership competencies as a part of health practitioner education. Published evidence, both global and Indian, was included in the literature search, looking for the need for interdisciplinary training of health practitioners, current scenarios in interprofessional health education, and key interdisciplinary leadership competencies that could be incorporated into the curricula of health practitioners. The literature search strategies included journal articles from electronic databases, medical, nursing and public health journals, gray literature, newspaper articles, and papers presented at conferences. The search was conducted around three thematic areas: (i) general leadership competencies in healthcare, (ii) specific leadership competencies in medical, nursing and public health, and (iii) interdisciplinary leadership competencies. Search terms included “leadership,” “leadership competencies,” “leadership in health care,” “leadership in medical education,” “leadership in nursing education,” “leadership in public health education,” “interprofessional education,” and “interdisciplinary leadership.” These were used individually as well as in combination. The search was not restricted by the period of publication or language. The electronic search was complemented by hand searching for relevant publications/documents in bibliographies. A process of snowballing was used until no new articles were located. Once the articles were procured, each article was reviewed by a team member for its relevance to the project with appropriate data extracted. The working group subsequently summarized the findings of the search and prepared a formal report, which was reviewed by all the members and then finalized. This was followed by a consultation with experts from various disciplines of health professional education, where the findings of the literature search were considered.

The next activity of the Collaborative was the development of a training model to be tested subsequently as pilot training sessions. The draft training model was conceptualized based on the findings of the literature search, deliberations, and the recommendations of the expert group at the consultation and other representatives from the three professions. A detailed training manual was developed for use in pilot training by the working group along with the team leads. The long-term objective of this training model was its integration into the regular curriculum of the medical, nursing, and public health programs, with the aim of developing interdisciplinary leadership skills relevant to twenty-first century health system challenges. This was deemed necessary for a positive change in the healthcare system of India, with inclusion of interdisciplinary leadership competencies in the health practitioners’ education curricula.

In alignment with the objectives of the IC, the training model was pilot-tested on medical, nursing, and public health practitioners and students across the three professions. A detailed agenda was prepared based on the content of the training manual. The pilot training sessions, held in 2013, were conducted in three clusters.

## Outcomes of the Activities

### Literature search

The literature search relating to leadership in the context of healthcare and interdisciplinary leadership competencies yielded abundant information on the subject. It was observed that leadership and management are essential for optimal performance in healthcare, bolstering service delivery and improving outcomes in health systems. Hospitals with improved leadership and management practices experience superior clinical performance, stronger financial positions, and higher patient satisfaction (8). Leadership includes developing the vision of the organization, empowering staff and management, establishing organizational culture and values, and understanding the characteristics of leaders and communication (9).

The current medical curriculum requires restructuring, with an increased focus on key competencies in several domains of public health. Currently, a majority of physicians lacks the technical skills necessary for major leadership/management roles that will enable them to both change and empower the local healthcare service delivery environment (10). Therefore, significant training in leadership skills as part of the medical curriculum is important.

The majority of current nursing education involves acute care while incorporating the skills necessary to negotiate with the healthcare team. As a result, nurses need to attain the requisite competencies to deliver high-quality care, including leadership, health policy, system improvement, research and evidence-based practice, and teamwork and collaboration. While the program for Auxiliary Nurse Midwives in India has limited scope for decision-making, some of the other programs include leadership education in which the staff nurses are empowered to make decisions, manage wards, and delegate assignments.

Over the years, public health education has largely been restricted to medical colleges as part of the medical undergraduate curriculum. However, lately, a conscious shift in public health education in India has occurred with institutions offering public health programs for medical as well as non-medical graduates, thereby widening the scope of public health education across practitioners from diverse fields. While these practitioners are trained in core public health, training in leadership skills is lacking. The leadership competencies expected from practitioners of each of these three programs are found in Table 1.

**Table 1:** **Literature review of key leadership competencies for healthcare practitioners**.

Medicaldoctors (10–16)	Nursing practitioners (17)	Public health practitioners (18)
Emotional intelligence, confidence, humility and creativity as necessary qualities of leaders, teamwork, communication, management, quality improvement, strategic and tactical planning, persuasive communication, negotiation, financial decision-making, team building, conflict resolution, and interviewing	Designing and implementing plans for care of the sick, evidence-based practice, population-appropriate healthcare, clinical decision- making, risk anticipation, accountability for evaluation and improvement of point-of-care outcomes, client and community advocacy, delegation and oversight of care delivery and outcomes, team management and collaboration with other health professional team members, and development and leveraging of resources	Visionary leadership skills, effective change agent, political prowess, negotiation and mediation, ethics, marketing and education, organizational capacity and dynamics, transorganizational collaboration, social forecasting, and team building

Improved patient care and population health with reduced cost is a primary target of the modern healthcare system. Health practitioners with different professional backgrounds come together to attain a common goal – patient care. Health systems, patients, and providers benefit when collaboration is practiced. Environments that encourage collaborative partnerships require strong leadership. Interdisciplinary leadership competencies are increasingly essential in the international healthcare environment. Interdisciplinary leadership has emerged from traditional models of leadership that are obsolete in the health reform environment. Leadership styles are often discussed based on behaviors used to influence change. Over the years, several models of interdisciplinary leadership such as transactional, collective, transformational, renaissance, quantum, pluralistic, post-heroic, servant, Zen, and other leadership models have emerged (19, 20).

### Interdisciplinary leadership competencies

Based on the literature search and the deliberations during the consultation that followed the search, the following core interdisciplinary leadership competencies were identified:

- Be self-aware
- Visionary with a sense of mission
- Self-regulation
- Committed and motivated
- Decisive, courageous, and honest
- Good communication/interpersonal skills
- Influence peers to innovate
- Strategic and tactical planning
- Networking, team collaboration
- Encourage innovation and facilitate transformation
- Set a direction
- An effective change agent and role model

In order to arrive at a common set of competencies for all the three professions, the research team entered into discussions with experts, representatives from the three professions as well as reviewed the literature to identify the functions for these three professions. The core group reviewed the job responsibilities and functions and collated all the functions identifiable as a leadership function. The duplicates were removed and similar functions were merged to build a common list of leadership-related responsibilities. We presented these for suggestions to the technical advisory group and incorporated their suggestions to evolve a set of core leadership competencies. These competencies guided us to develop a training manual incorporating learning objectives that best reflected these competencies.

### Interdisciplinary leadership training

The literature review findings formed the basis for developing a training model, which was subsequently piloted at three different sites: State Institute of Health Management and Communication, Gwalior (SIHMC); Indian Institute of Public Health, Bhubaneswar (IIPHB); and Datta Meghe Institute of Medical Sciences, Sawangi (DMIMS). The duration of each training session was 3 days. Selectively chosen senior practitioners based in the partner institutions served as resource faculty for the pilot training, actively engaging the participants. The training workshop groups were a mix of participants from different disciplines. The total number of participants across the three cohorts was 66, 26 of which were females. The average age of the participants across all groups was 32 years.

The pilot training workshops included didactic sessions as well as group discussions. The didactic sessions were aimed at giving the trainees an understanding of leadership skills and their importance in healthcare. The group discussions aimed at training them to innovatively apply interdisciplinary leadership competencies in their local healthcare settings. The agenda of the sessions was designed to coalesce the groups so as to form a team with interdisciplinary leadership skills. Details of the training model with topics covered are provided in Table 2.

**Table 2:** **Agenda and pedagogy of the pilot interdisciplinary leadership training**.

Timings	Activity	Pedagogy
**DAY 1**		
0930 to 1300	Introductions and sharing of leadership training experiences	Each participant was given a card to note down their leadership experiences and expectations, followed by introductions and a brief overview of the program by the training coordinator
	Objectives of training program	
	Program schedule	
	Expectations of participants	
	Project background: interdisciplinary leadership skills among health practitioners in the twenty-first century	
1300 to 1400	Lunch
1400 to 1700	What is leadership?	These sessions were didactic, followed by group discussions
	Characteristics of leadership	
	Leadership styles	
**DAY 2**		
0930 to 1300	Interview with guest speaker (focus on leadership qualities and challenges), Discussion	A senior health professional, who has previously worked in a leadership position, was invited and interviewed by some participants. Questions during the interview included his experiences as a leader, challenges he faced, coping mechanisms, etc.
1300 to 1400	Lunch	
1400 to 1700	Motivation	Case studies discussed with the participants
	Time management	Didactic session followed by group discussion
**DAY 3**		
0930 to 1300	Managing an organization	Role play, followed by group discussion
	Team building	A movie highlighting the importance of team building was shown to the participants, followed by a group discussion
1300 to 1400	Lunch break	
1400 to 1500	Concluding session	Each participant was asked to correlate his/her expectation from the first day and what was learnt during the 3-day training and give general feedback regarding the training

At the end of each of the training sessions, the trainees were asked to provide feedback regarding various aspects of the program. Many positive responses from the participants were received, ranging from good coordination of the training, beneficial content, diverse and relevant pedagogy, to a friendly atmosphere. A few negative points, such as short duration of the training, theoretical sessions, and less group discussions/practicum were also emphasized in the responses. Based on the feedback of the trainees, the training model was revised. The duration of the training was increased to 4 days. Certain topics, such as Ethics of Leadership, Advocacy, Conflict Resolution, Negotiation, and Interpersonal Communication, were added to the program. The program was revised to include more group discussions, role playing, videos, and innovative themes such as the World Café. The revised model was shared with members of the Technical Advisory Group and finalized after their input.

Following the structure adopted for the pilot training and incorporating the lessons learned from them, further rounds of training workshops on leadership and its relevance in the health and development sectors continue to be conducted regularly to propagate the agenda of the IC. Participants of these training workshops include practitioners from the health and development sectors, such as medical, nursing, program management, public health organizations, academicians, consultants, and practitioners from the industrial sector.

## Way Forward

Leadership is not only about being seen as the leader but also about developing the personal qualities required to work effectively with others, hence learning to work within teams and developing followership skills are essential values of interdisciplinary leadership (21). In the field of healthcare in India, interdisciplinary leadership is still at a nascent stage. The current curricula for medical, nursing, and public health education do not adequately provide for formal training in individual or interdisciplinary leadership skills. Although healthcare is a sector where interdisciplinary teams are expected to function to serve a common goal – patient care, it is very often observed that the medical, nursing, and public health professions tend to work in silos, with one group tending to overpower the others. The *Future of Nursing* Report recommends a strong and committed partnership of nursing practitioners with physicians and other health practitioners in building leadership competencies to develop and implement the changes required to increase quality, access, and value and deliver patient-centered care (3). Health systems, patients, and providers benefit when collaboration is practiced. Healthcare organizations that have competent clinical leaders tend to have greater staff engagement, better performance, and higher quality of care with improved outcomes (22).

### Need for leadership skills

Increasing coverage of priority health services requires additional resources and good leadership is vital to using these resources effectively to achieve measurable results for positive system change (23). Primary healthcare demands equal participation and responsibility from all team members with leadership shifting between practitioners determined by the nature of the problem to be solved. Although medical education may be the most suitable opportunity for building leadership skills among physicians, the nursing and public health education systems must also be considered for reforming their respective curricula. This provides an opportunity to incorporate leadership competencies in the curricula for the development of health leaders (14). In times of change, a leader is needed, who can take initiative and responsibility, influence many different disciplinary groups, design new ways of working, be a pioneer, and follow a different and vibrant vision. McKinsey and Company states that leadership development can fundamentally change the way health systems work, provided the basic four principles of leadership are adhered to: (i) leadership development must form the backbone of a health system transformation, not merely serve as its complement, (ii) it must follow an overarching plan, (iii) it must strengthen who leaders are, not just what they do, and (iv) senior system leaders must sit in the center of leadership development, spearheading the leadership development effort, and not sit on the sidelines (24).

### Interdisciplinary leadership in the indian context

Interprofessional team leadership is a prerequisite in healthcare settings as much in India as it is globally. For this, continuous efforts on the part of multiple stakeholders are vital for transforming the current status by creating and testing different models of interprofessional practice. Interprofessional education, training, and practice can make a positive difference by promoting a reflective practice environment that can generate and encourage shared knowledge (4). Achieving leadership skills by “work experience” or “on the job” is demanding. Since leadership is deemed to be relevant at all levels, leadership development must be addressed throughout the education and training undertaken by health practitioners – medicine, nursing, and public health, and sustained on the job.

Leadership skills are not yet being optimally included in health professional education in India. Leadership competencies, although essential, compete with the requirement for higher clinical competencies within the curricula, thereby posing a challenge in the acquisition of leadership competencies. Unlike United States, where the Core Competencies for Interprofessional Collaborative Practice suggests interprofessional team building and leadership as one of 11 identified competency area for clinicians, there is no such contextual document that outlines interdisciplinary leadership competencies in India (5). There is limited experience even in the assessment of such interdisciplinary competencies. Carlton et al. have suggested a model for assessing interprofessional competency development adapted to non-clinical professions (7). Although they have not used this model specifically for non-clinical competencies and not for leadership competencies, there is potential for using this model to assess interdisciplinary leadership competencies for health professionals.

The actual needs and current demands of the public health systems in India and most developing countries are at odds with embracing these educational reforms. At present, the health professional education curriculum in India does not include leadership competencies to strengthen health systems and to address the health challenges of the new century. A framework of leadership competencies for health professional education in the Indian context may be drawn from global knowledge and experiences but must be relevant to local context. India needs an empirical model of leadership competencies for health professional education that can be employed to further interdisciplinary leadership within the complexities integral to the health system.

### Recommendations

To comprehensively address these leadership issues, healthcare requires significant reforms in health professional education. Competencies such as self-awareness, self-regulation, commitment, motivation, enthusiasm, empathy, social skills, decisiveness, courage, and integrity emerge as universal set of requirements, which can be learned and developed. While these skills are mainly in use in management circles currently, they should prove to be central and beneficial to the healthcare organization’s strategic action plan. However, further research is warranted to validate the role of these interdisciplinary competencies. Potential areas for research can include impact of the introduction of this leadership training on improving organizational and population outcomes. Since these competencies are contextualized for interdisciplinary work, further research is desirable to assess sustainable individual and organizational gains in case of changes in the team composition within the healthcare settings. We have assumed that a common set of leadership competencies is applicable across all these three professions. While a case for a common set of leadership competencies for doctors and nurses in clinical care may be easier to accept, there is a greater divergence of functions of public health professionals within countries.

Although the duration of the training conducted was short, it was well received by the participants. The pilot training demonstrated the need for interdisciplinary leadership competencies in the field of healthcare. Although at an embryonic stage, the demand for additional training reflects the desire among the younger healthcare practitioners for collaborative leadership. The current lack of progress in advancing interprofessional collaboration strongly suggests that academic and health practitioners’ reluctance in accepting that concept that interprofessionalism will improve coordination of health services, better use of specialist resources, and provide improved health outcomes. When the benefits provided through interprofessional collaboration are more widely accepted, then a breakthrough may be reached (4). Formal incorporation of leadership skills in health professional education is possible within interdisciplinary classroom settings using the principles of transformative learning. The adoption of a case-based learning that encourages participation of students from diverse disciplines has a potential for enhancing acquisition of interdisciplinary competencies (7) and needs further examination in the leadership context.

## Ethical Considerations

The authors did not interview or collect any data from the participants as a part of this activity. This activity only included undertaking a literature search and piloting of short 3-day interdisciplinary leadership training workshops by developing training materials. There was no component of primary research that was planned as part of the work. Therefore, an ethics approval was not required for this activity.

## Author Contributions

PN, HN, KS, and SZ contributed to the design and development of the project; collection, compilation, and interpretation of data for the project; drafting the article and revising it for important intellectual content; finalization of the version to be submitted for publication; and agree to be accountable for the article. RT contributed to collection and compilation of data; drafting the article; approval for finalization of version to be submitted for publication; and agree to be accountable for the article. ZQ, AG, JN, MG, and RY contributed to the design and development of the project; collection and compilation of data; drafting the article; approval for finalization of version to be submitted for publication; and agree to be accountable for the article.

## Conflict of Interest Statement

The authors declare that the research was conducted in the absence of any commercial or financial relationships that could be construed as a potential conflict of interest.
